# CPR Guidance by an Emergency Physician via Video Call: A Simulation Study

**DOI:** 10.1155/2018/1480726

**Published:** 2018-11-29

**Authors:** Dong Keon Lee, Seung Min Park, Yu Jin Kim, Choung Ah Lee, Won Jung Jeong, Gi Woon Kim, Dong Hyuk Shin, Young Hwan Lee

**Affiliations:** ^1^Department of Emergency Medicine, Seoul National University Bundang Hospital, Gyeonggi-do, Republic of Korea; ^2^Department of Emergency Medicine, Dongtan Sacred Heart Hospital, Hallym University School of Medicine, Hwaseong, Republic of Korea; ^3^Department of Emergency Medicine, The Catholic University of Korea, St. Vincent's Hospital, Suwon, Gyeonggi-do, Republic of Korea; ^4^Department of Emergency Medicine, College of Medicine, Soonchunhyang University, Bucheon, Gyeonggi-do, Republic of Korea; ^5^Department of Emergency Medicine, Kangbuk Samsung Hospital, Sungkyunkwan University School Medicine, Seoul, Republic of Korea

## Abstract

**Background:**

In South Korea, the prehospital treatment of cardiac arrest is generally led by an emergency medical technician-paramedic (EMT-P), and defibrillation is delivered by the automatic external defibrillator (AED). This study aimed at examining the effects of direct medical guidance by an emergency physician through a video call that enabled prompt manual defibrillation.

**Methods:**

Two-hundred eighty-eight paramedics based in Gyeonggi Province were studied for four months, from July to November 2015. The participants were divided into 96 teams, and the teams were randomly divided into either a conventional group that was to use the AED or a video call guidance group which was to use the manual defibrillators, with 48 teams in each group. The time to first defibrillation, total hands-off time, and hands-off ratio were compared between the two groups.

**Results:**

The median value of the time to the first defibrillation was significantly shorter in the video call guidance group (56 s) than in the conventional group (73 s) (p<0.001). The median value of the total hands-off time was also significantly shorter (228 vs. 285.5 s) (p<0.001), and the hands-off ratio, defined as the proportion of hands-off time out of the total CPR time, was significantly shorter in the video call guidance group (0.32 vs. 0.41) (p<0.001).

**Conclusion:**

Physician-guided CPR with a video call enabled prompt manual defibrillation and significantly shortened the time required for first defibrillation, hands-off time, and hands-off ratio in simulated cases of prehospital cardiac arrest.

## 1. Introduction

In South Korea, the rates of restoration of spontaneous circulation (ROSC) following cardiac arrest in 2012-2015 were 5.1% and 23.1% before and after hospital arrival, respectively, while the rate of survival to hospital discharge among patients who were hospitalized after the acute cardiac arrest was merely 5.0% [1]. Further, the proportion of patients who were discharged with the good neurological outcome (cerebral performance categories 1, 2), which is the final resuscitation goal for out-of-hospital cardiac arrest patients, was only 2.3%. This is an extremely low rate compared to the rate of 6.9% reported in the U.S. nationwide CARES study from 2005 to 2010 and 8.9% reported in a Japanese study conducted in Osaka from 2007 to 2009 [2, 3].

In South Korea, as physicians are not staffed in 119-ambulances, the prehospital treatment is generally led by an emergency medical technician-paramedic (EMT-P) and manual defibrillator use, advanced airway insert, and intravenous line placement are not allowed to EMT-Ps without the physician's direct medical control in point of law.

In the prehospital cardiac arrest, physician-guided CPR is controversial as to whether there is survival benefit compared to paramedic-guided CPR. However, some studies reported that during CPR, the presence of physicians had been reported to increase compliance with the guidelines that could lessen the hands-off time [4–9]. Moreover, in a simulation study by Pytte et al., both the times from the last compression to defibrillation and from defibrillation to chest compression were shorter with manual defibrillators than with AEDs, which was also supported by a retrospective study on actual patients by Kramer-Johansen et al. [10, 11].

Taken together, these studies indicate that prolonged pause of chest compression reduces the coronary and cerebral perfusion pressure, which may have deleterious consequences for the patient.

Recent advances in video call technology have enabled remote monitoring of cardiac arrest status through smartphones. One previous study that examined the differences in the quality of dispatcher-assisted chest compression between video coaching and audio coaching and the video coaching showed better results regarding the compression rate, the accuracy of the compression site, and the minimisation of the hands-off time [12].

Based on these previous studies and the current situation in South Korea, the present study aimed at examining the effects of direct medical guidance by an emergency physician through a video call and the effect of the manual defibrillator use.

## 2. Methods

### 2.1. Study Participants and Design

**Figure 1:**
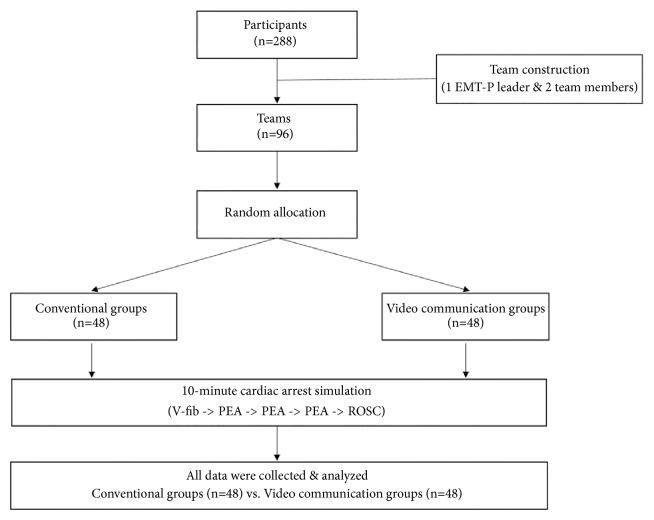
Flow diagram of the study.

Two-hundred and eighty-eight paramedics based in Gyeonggi-do Province were studied for four months, from July to November 2015. The participants were divided into 96 teams of one EMT-P leader and two team members, and the teams were randomly divided into either a group using the AEDs (conventional group) or a group using manual defibrillators with video guidance by a physician (video call guidance group), with 48 teams in each group (Figure 1).

Ninety-six teams of EMT-P were trained for eight hours on video-guided CPR in theory and practice. The training was conducted for three months, considering the working hours of paramedics. After training, the simulations were conducted four times a day for a month at the simulation centre in a tertiary university hospital located downtown and video-recorded using an SMX-F34 camcorder (Samsung, South Korea), with consent from the trainees. The ALS Simulator (Laerdal, Norway) and Biphasic defibrillator-monitor Dixion HD-1 (Dixion, Germany) were used for the simulations.

**Figure 2:**
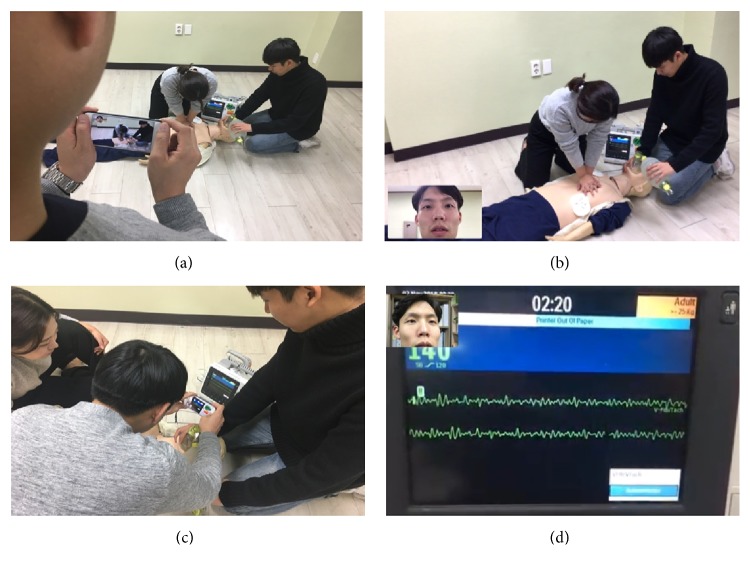
The video call for the physician's real-time direct medical control. (a) The EMT-P leader held the phone to show the overall situation to the guiding physician. (b) The guiding physician's view of the overall situation. (c) The EMT-P leader shows the cardiac rhythm on the defibrillator to the physician, the overall situation to the guiding physician. (d) The guiding physician's view of the cardiac rhythm on the defibrillator.

In the simulation scenarios, EMTs were dispatched after receiving a call for cardiac arrest. The participants in the conventional group performed cardiopulmonary resuscitation with AED, and the members in the video call guidance group connected the video call just before scene arrival to perform cardiopulmonary resuscitation with manual defibrillators with a physician's real-time direct medical control via the video call. For the video call, one EMT-P leader held the phone to communicate with the guiding physician and show the cardiac rhythm on the defibrillator, the overall situation to the guiding physician (Figure 2). Galaxy S6 (Samsung, South Korea), which supports long-term evolution (LTE), was used for a video call connection.

A total of six emergency physicians were involved in this study. One guiding physician participated per scenario, and the cardiac rhythm was analyzed every two minutes. Ninety-six clinical case scenarios were done, which had the same 10-minute simulation of the following protocol: ventricular-fibrillation -> pulseless electrical activity (PEA) -> PEA -> PEA -> ROSC. Two persons other than the guiding physicians manipulated the cardiac rhythms so guiding physicians could be blinded to the cardiac rhythm flow. The role of the guiding physician involved was to guide the trainees in using the manual defibrillator according to the arrest rhythm and encourage them to shorten the hands-off time. The EMTs and guiding physicians were in different rooms and only communicated via video call.

### 2.2. Outcome Measures and Assessment

The primary outcomes were the time from arrival to the first defibrillation and the hands-off ratio. Secondary outcomes included the time from the last compression to defibrillation and the time from defibrillation to chest compression. To ensure consistency in the assessments, two advanced cardiovascular life support instructors who were not involved in the study assessed and scored each item while viewing the recorded video.

### 2.3. Statistical Analysis

Categorical data are expressed as the number and frequency. Continuous data are presented as the mean and standard deviation, median with interquartile range, or mean and range. Differences between the two groups were tested using the independent two-sample t-test or Mann–Whitney U test for continuous variables, and the chi-square or Fisher's exact test was used for categorical variables. p < 0.05 was considered significant. SPSS software (version 18.0; SPSS Inc, Chicago, IL, USA) was used for all analyses.

Statistical power was calculated using G*∗*Power 3.1, and the primary outcome was defined as time to first defibrillation (seconds). Mean time to the first defibrillation showed 81, 61, and standard deviations were 33.1, 24.3 in the conventional group and video call guidance group, respectively. Power was calculated as 0.948 based on an effect size d of 0.688162 and alpha error probability of less than 0.05.

## 3. Results

The two groups of 48 teams were analyzed for their background characteristics. There were no significant differences in the sex, age, and years of work experience between the two groups (Table 1) and the appropriateness of defibrillation was 100% for both groups (Table 2). The median value of the time to the first defibrillation was significantly better in the video call guidance group (56 s) than in the conventional group (73 s) (p<0.001). The median value of the total hands-off time was also significantly better (228 vs. 285.5 s) (p<0.001), and the hands-off ratio, defined as the proportion of hands-off time out of the total CPR time, was significantly shorter in the video call guidance group (0.32 vs. 0.41) (p<0.001) (Table 2).

In terms of the secondary outcomes, the median value of the time from the last compression to defibrillation was significantly shorter in the video call guidance group (3 [IQR 2–7] s) than in the conventional group (8 [IQR 3–19] s) (p<0.001), as was the median value of the time from defibrillation to resumed chest compression (2 [IQR 1–3] vs. 3 [IQR 2–4] s) (p<0.001) (Table 2).

## 4. Discussion

This study investigated the effects of physician-guided CPR via video calls and the use of manual defibrillators in out-of-hospital cardiac arrest situations where an emergency physician is not dispatched. Our results showed that the video call guidance group had significantly better performance in terms of the time to first defibrillation, hands-off time, hands-off ratio, time from the last compression to defibrillation, and time from defibrillation to resumed compression.

South Korea has a considerably lower rate of successful resuscitation by CPR than other developed countries, such as the United States and Japan, with one reason being the low success rate of prehospital resuscitation. Considering that the average time for the paramedics to arrive at the scene is about 7-8 minutes, the successful resuscitation rate should be about 6-7% [1], highlighting the need to improve the quality of prehospital CPR. As one measure to address this issue, studies have sought to improve the quality of bystander CPR by facilitating communication between the dispatcher and bystander using voice and video calls, with video-assisted communication showing superior outcomes over voice calls [13, 14]. According to a study by Yang et al., the group using video calls showed better outcomes regarding securing and maintaining open airways, appropriate chin lift, and ventilation volume [13]. Moreover, one previous study reported that having eye-contact with a dispatcher through a video call and being supervised and directed by the dispatcher increased the confidence of lay rescuers in highly stressful emergency situations [14]. Tränkler et al. investigated whether it was possible to obtain an adequate understanding of the CPR scene through a 3G video call and concluded that 3G video calls were indeed sufficient in relaying accurate information about the CPR scene with appropriate distance between the camera and object, angle, and lighting [15]. However, these studies were all conducted in the general population, and, to date, no existing study has examined paramedic performance under direct physician guidance using long-term evolution video calls. In this study, the video call guidance group showed significantly better performance in the time to first defibrillation (56 s) than the conventional group (73 s) (p<0.001). These positive results support the recent CPR guidelines in which the importance of performing defibrillation as quickly as possible is stressed [16] and indicate that dispatchers can provide quicker analysis and directions than AEDs.

The longer the hands-off time is, the more the blood flow to the heart and brain is reduced, and the lower the probability of ROSC is, suggesting that chest compression pauses should be kept at a minimum [17, 18]. Currently, manual defibrillators are legally prohibited for use by the Emergency Medical Services in South Korea. In our study, an emergency physician confirmed the rhythm of the manual defibrillators and directed defibrillation via a video call, and the group that received such directions showed superior performance compared to the conventional group in the hands-off time, and hands-off ratio, corroborating the results of previous studies that compared CPR using AEDs and manual defibrillators [10, 11]. Furthermore, in our study, the time from last compression to defibrillation and the time from defibrillation to resumed chest compression were significantly shorter in the video call guidance group, which is speculated to be due to the fact that the guiding emergency physician could quickly analyze the rhythms and continuously encouraged the paramedics to maintain constant chest compression.

This study has some limitations that need to be acknowledged. First, the quality of video calls may vary according to the LTE network quality and environmental factor; however, we did not investigate these differences in the present study.

Second, there may be differences between the simulated environment and the real situation. Third, since there are many kinds of AEDs and manual defibrillators, the outcome might be different from other kinds of defibrillators.

## 5. Conclusions

Physician-guided CPR with a video call enabled prompt manual defibrillation and significantly shortened the time required for first defibrillation, hands-off time, and hands-off ratio in simulated cases of prehospital cardiac arrest. These results should be verified in real clinical situations in the near future.

## Figures and Tables

**Table 1:** General characteristics of the study groups.

	**Conventional group**	**Video call guidance group**	***p*-value**
Male sex, n (%)	97 (67.4)	102 (70.8)	0.85
Mean age, years (range)	32 (25 ~ 43)	33 (27 ~ 42)	0.89
Ethnicity			
Asian, n (%)	144 (100%)	144 (100%)	
Body Mass Index, kg/m^2^ (mean ± SD)	23.4 ± 5.1	23.9 ± 4.8	0.79
Working experience, years (mean ± SD)	3.7 ± 1.5	3.6 ± 1.3	0.72
BLS provider, n (%)	134 (93.0)	130 (90.3)	0.88

SD: standard deviation, BLS: basic life support.

**Table 2:** Cardiopulmonary resuscitation performance.

	**Conventional group** **(48 teams)**	**Video call guidance group** **(48 teams)**	***p*-value**
Accurate D/C shock			1.00
Yes	48 (100%)	48 (100%)	
No	0 (0%)	0 (0%)	
Time to 1st defibrillation (seconds)	73 (59.75~90)	56 (44~74.25)	<0.001
Time from last compression to defibrillation (seconds)	8 (3~19)	3 (2~7)	<0.001
Time from defibrillation to chest compression (seconds)	3 (2~4)	2 (1~3)	<0.001
Total CPR time (seconds)	704.5 (674~740)	686.5 (663~706.2)	0.004
Total hands-off time (seconds)	285.5 (299.75~339.0)	228 (192.75~270.25)	<0.001
Hands-off ratio (total hands-off time/total CPR time (seconds)	0.41 (0.33~0.47)	0.32 (0.27~0.38)	<0.001

The values (except accurate D/C shock) are presented as the median (interquartile range).

BLS: basic life support, D/C: direct current, CPR: cardiopulmonary resuscitation.

## Data Availability

No data were used to support this study.
